# Self-mastery buffers associations between stressful life events and mental quality of life and fatigue in people living with multiple sclerosis

**DOI:** 10.1007/s11136-026-04287-9

**Published:** 2026-06-06

**Authors:** Maggie Yu, Steve Simpson-Yap, George Jelinek, Sandra Neate, Jeanette Reece, Nupur Nag

**Affiliations:** 1 Neuroepidemiology Unit, Centre for Epidemiology and Biostatistics, Melbourne School of Population and Global Health, The University of Melbourne, Melbourne, VIC Australia; 2 The Florey Institute of Neuroscience and Mental Health, The University of Melbourne, Melbourne, VIC Australia; 3 MS Research Flagship, Menzies Institute for Medical Research, University of Tasmania, Hobart, TAS Australia

**Keywords:** Mastery, Stressful life events, Quality of life, Fatigue, Multiple sclerosis

## Abstract

**Background:**

Multiple sclerosis (MS) is a chronic immune-mediated inflammatory disease of the central nervous system, often accompanied by fatigue and reduced quality of life (QoL). Stressful life events (SLEs) may heighten stress in people with MS (pwMS), compounding the challenges of living with the condition. While mastery is acknowledged as a psychological resource for resilience, its protective role against SLEs remains unclear. We examined whether mastery modifies associations between SLEs and QoL and fatigue in pwMS.

**Methods:**

We analysed cross-sectional survey data from an international sample of 948 pwMS. Mastery (Pearlin mastery scale) was categorised into tertiles (low, moderate, high). QoL (MSQoL-54) was summarised as mental and physical composite scores. Clinically significant fatigue was defined as fatigue severity scale (FSS) > 5. SLEs exposure (Holmes–Rahe social readjustment rating scale) was assessed as total number and load (severity). Multivariable regression models assessed associations between SLEs and outcomes, and interaction terms tested effect modification by mastery.

**Results:**

Higher number and load of SLEs were associated with clinically meaningful lower mental QoL (− 7 to − 8 points) among pwMS with low or moderate mastery, but not among pwMS with high mastery. Higher number and load of SLEs were also associated with 3– 5 fold higher odds of clinically significant fatigue, primarily among pwMS with low mastery.

**Conclusion:**

Higher mastery may buffer adverse associations between SLEs exposure and mental QoL and fatigue. Prospective studies are needed to confirm temporal relationships; however, interventions that support mastery may help mitigate stress-related impacts on wellbeing in MS.

## Introduction

Multiple sclerosis (MS) is a chronic immune-mediated inflammatory disease of the central nervous system, predominant in females [1], that can cause a wide range of physical, cognitive, and psychological symptoms [2, 3]. These symptoms, alongside the burden of ongoing treatment and self-management, contribute to substantially reduced health-related quality of life (QoL) in people with MS (pwMS) [4, 5]. QoL is therefore a key outcome for clinical care.

Stressful life events (SLEs) are major life occurrences, such as marriage, divorce, death or illness of a family member, or job transitions, that require significant adjustments and can increase levels of stress [6]. PwMS, facing SLEs and the challenges of living with MS, often experience high stress and adverse health conditions [7, 8]. In a study of 109 pwMS, nearly 90% reported experiencing stress, with 48% indicating high-stress levels [9]. A recent systematic review of 32 studies reported that 22% of pwMS have severe anxiety, significantly exceeding the prevalence seen in the general population [10].

QoL, a key indicator of the impact of symptoms and treatments, is significantly lower in pwMS than in individuals with other chronic illnesses or the general population [4, 11]. Research on SLEs has focused on their impact on onset of MS and of relapses [8, 12], with limited exploration of their impact on QoL. One study, utilising the perceived stress scale on 10 life situations, found that higher perceived stress in pwMS was associated with lower scores across all domains of QoL [9]. A few studies have shown the negative effect of SLEs on QoL in cancer patients. For instance, in a cohort of 662 cancer patients, the total number of SLEs in the past 12 months was associated with significantly lower mental (mQoL) and physical QoL (pQoL) [13]. Another study involving 152 patients with chronic lymphocytic leukemia found that perceived stress from major events was associated with a 17% decrease in mQoL over a 5-month period, but no association was found with pQoL [14].

Fatigue is among the most distressing and functionally limiting symptoms of MS and affects up to 80% of pwMS [15]. While associations between SLEs and fatigue have been reported in other chronic conditions [14, 16], evidence in pwMS remains limited and mixed. A 2-year prospective study found number and perceived severity of SLEs, measured by the life-event stressor scale, were linked to increased fatigue in 80 pwMS [17]. Likewise, a cross-sectional study with 1,239 pwMS showed higher number of SLEs was associated with 7–15% higher fatigue [18]. However, our previous cross-sectional study, which included 948 pwMS from an international cohort, did not find an association between either number or load (severity) of SLEs with fatigue [19]. These inconsistent findings suggest that individual resilience resources may influence how stress exposure translates into symptom burden and QoL outcomes.

Mastery, which refers to one’s sense of control over life circumstances, has been recognised as a psychosocial trait that promotes resilience to stress [20, 21]. In pwMS, higher mastery has been associated with more adaptive coping behaviours and lower perceived stress [22, 23]. In other populations, including the general population and people living with HIV, mastery has been shown to buffer the adverse effects of SLEs on wellbeing [24, 25]. However, whether mastery modifies associations between SLEs exposure and QoL or fatigue in pwMS is unclear.

In this study, we examine associations between SLEs exposure (number and load) and QoL and fatigue, and test whether these associations differ by mastery level. If mastery buffers the impact of SLEs, interventions that strengthen mastery may represent a practical target to mitigate stress-related decrements in QoL and fatigue in pwMS.

## Materials and methods

### Participants

De-identified data from the health outcomes and lifestyle in a sample of pwMS (HOLISM) study [26] were analysed. Data was restricted to participants aged ≥ 18 years, self-reporting a physician-confirmed MS diagnosis, who completed the SLEs survey queried only at 7.5-year follow-up. Ethics approval was granted by The University of Melbourne Human Research Ethics Committee (ID1545102).

### Instruments

#### Sociodemographics and clinical variables

Participants completed an online survey capturing sociodemographic and clinical information, including age, sex, highest level of education, perceived relative socioeconomic status (PRSES, queried from 2.5-year)[27], MS phenotype, ongoing symptoms due to recent relapse, duration since MS diagnosis, use of disease modifying therapies (DMTs), and use of prescription medications for depression and fatigue.

Disability was assessed using the patient-determined disease steps (PDDS) [28] from which the disease-duration-adjusted Patient-determined MS Severity Score (P-MSSS) was estimated [29]. P-MSSS was categorised to normal/mild (0–3), moderate (> 3–6), and severe (> 6). Depression was queried via PHQ-2 and defined as PHQ > 2 [30]. Treated comorbidities were queried by self-administered comorbidity questionnaire (SCQ) [31] and total number categorised to 0, 1, and ≥ 2. Number of social support persons was queried by selection of number (0, 1, 2–5, 6–9, ≥ 10) [32] and categorised as 0–1, 2–5 and ≥ 6.

#### Stressful life events

SLEs were queried via a modified Holmes–Rahe social readjustment rating scale [6], where 16 SLEs were included based on a previous study assessing associations between SLEs and MS onset [33]. Participants reported whether they had experienced any of the 16 SLEs (e.g., death of significant other, major financial crisis, gained a new family member) in the preceding 12 months.

SLEs number and load variables were derived. SLEs number is a summation of SLEs, value range 0–16, then categorised into 0, 1, and ≥ 2. SLEs load refers to the stress level related to SLEs, calculated by numerically weighting (value range 0–100) each SLEs and summating the weighted SLEs [34]. For example, death of a significant other is weighted “100”, while ‘a crisis or disappoinment in work, school or career’ is weighted “22”. SLEs load was categorised into quartiles.

Participants rated a positive or negative impact for each SLEs using a scale of − 5 to + 5. Analyses thus also included associations with negative SLEs number and load (restricted to events perceived as negative by participants). Four SLEs variables were utilised for analysis in this study: total SLEs numbers, total SLEs load, negative SLEs numbers, and negative SLEs load [19].

#### Outcome variables

QoL was queried using the MSQoL-54 [35], comprising 54 questions, each assessed via a 5-point Likert scale. From these 54 questions, 12 subdomain scores were calculated, from which two composite scores for mQoL and pQoL were estimated, ranging from 0 (low) to 100 (high).

Fatigue was queried using the fatigue severity score (FSS), comprising nine questions, each scored with a 7-point Likert scale. Scores were averaged, a mean FSS > 5 defined as clinically significant fatigue [36].

#### Moderator variable

Mastery was assessed using the Pearlin mastery scale (PMS) [20], a 7-item questionnaire, each item assessed via a 4-point Likert scale. Five negatively worded items were reverse-coded and all items were summated to create a PMS total score ranging from 7 to 28. PMS total scores were categorised into tertiles for low (7–19), moderate (20–23), and high (24–28) mastery.

### Statistics

Data analyses were conducted in Stata/SE 17.0 (StataCorp, College Station, TX) with statistical significance set at *p* < 0.05. Differences in cohort characteristics between included vs. excluded participants were assessed by *t*-test and log-binomial regression for continous and binary/categorical variables, respectively.

Associations between SLEs and mQoL and pQoL were assessed by multiple linear regression models. Regression coefficients (β) and 95% confidence intervals (CI) were estimated. Associations between SLEs and fatigue was assessed using logistic regression, estimating odds ratios (OR) and 95% CIs. The moderation effect of mastery was assessed by adding interaction terms of mastery and SLEs variables into the models, results presented as SLEs-outcome by level of mastery along with a test for difference.

All models were adjusted for ongoing symptoms due to recent relapse, age, sex, MS phenotype, P-MSSS, number of treated comorbidities, PRSES, and number of social support persons. QoL models were further adjusted for fatigue and antidepressant medication use; fatigue models were further adjusted for depression and anti-fatigue medication use.

## Results

### Participant characteristics

Of 2,466 baseline participants, 948 (38%) completed the 7.5-year follow-up survey and were included in analyses (Table 1). The study population were predominantly female, with a mean age of 53 years. The majority held a university degree (71.3%) and had a non-progressive MS phenotype (72.5%). Approximately half of the participants perceived their SES as above average (53.5%), and a small proportion had only 1 or no social support person (22.3%). Most participants (56.2%) were of mild clinical disability (normal/mild disability (66.0%), clinically significant fatigue (41.0%), and no treated comorbidity (77.5%). Less than half of the participants used DMTs (44.2%), and small proportions reported depression (13.8%), used antidepressant (21.2%) or anti-fatigue medication (8.7%).

Compared to excluded participants (*n* = 1,518), the study population (*n* = 948) at baseline were more likely to be university educated, have more social support persons, report non-progressive MS phenotype, have higher mastery, mQoL and pQoL, and less likely to be overweight or obese, have moderate/severe disability, and have clinically significant fatigue and depression (Table 1).

**Table 1:** Characteristics of excluded vs included study participants

	Excluded participants (baseline)	Study participants (baseline)	Study participants (7.5-year)
	*N* = 1,518	*N* = 948	*N* = 948
	*n* (%)		*n* (%)
Demographics			
Sex			
Male	230 (16.1)	188 (19.8)	188 (19.8)
Female	1,196 (83.9)	760 (80.2)	760 (80.2)
University degree			
No	691 (45.8)	308 (32.6)	269 (28.7)
Yes	817 (54.2)	636 (67.4)^***^	668 (71.3)
PRSES			
Below average	na	na	170 (18.2)
Average	na	na	265 (28.3)
Above average	na	na	500 (53.5)
Support persons			
0–1	390 (29.6)	204 (22.6)	180 (22.3)
2–5	769 (58.3)	559 (61.8)^**^	499 (61.7)
≥ 6	160 (12.1)	141 (15.6)^***^	130 (16.1)
Clinical characteristics			
MS phenotype			
Non-progressive	912 (61.5)	679 (72.5)	679 (72.5)
Progressive	351 (23.7)	147 (15.7)^***^	147 (15.7)
Unsure/other	219 (14.8)	111 (11.9)^**^	111 (11.9)
Disability			
Normal/mild	740 (54.0)	598 (65.4)	615 (65.9)
Moderate	371 (27.1)	206 (22.5)^***^	206 (22.1)
Severe	260 (19.0)	110 (12.0)^***^	112 (12.0)
Fatigue			
No	614 (48.3)	517 (59.6)	506 (59.0)
Yes	657 (51.7)	350 (40.4) ^***^	352 (41.0)
Depression			
No	1,007 (76.5)	833 (87.3)	759 (86.3)
Yes	310 (23.5)	115 (12.7)^***^	121 (13.8)
Comorbidities			
0	802 (52.8)	573 (60.4)	735 (77.5)
1	355 (23.4)	219 (23.1)	100 (10.5)
≥ 2	361 (23.8)	156 (16.5)	113 (11.9)
DMTs use			
No	681 (44.9)	385 (40.6)	529 (55.8)
Yes	837 (55.1)	563 (59.4)	419 (44.2)
Antidepressant use			
No	1,173 (77.3)	791 (83.4)	747 (78.8)
Yes	345 (22.7)	157 (16.6)	201 (21.2)
Anti-fatigue medication use			
No	1,359 (89.5)	870 (91.8)	866 (91.4)
Yes	159 (10.5)	78 (8.2)	82 (8.7)

	Mean (SD)		Mean (SD)
Age, years	46.02 (10.61)	45.18 (10.25)	52.78 (10.24)
Mastery	20.6 (4.4)	21.7 (4.1) ^***^	21.3 (4.3)
mQoL	63.62 (21.82)	71.28 (19.75) ^***^	71.30 (20.70)
pQoL	55.42 (21.43)	64.39 (20.41) ^***^	63.80 (22.67)
SLEs number	na	na	2 (1–2)
SLEs number (negative)	na	na	1 (0–2)
SLEs load	na	na	57 (28–97)
SLEs load (negative)	na	na	46 (0–83)

DMTs, disease-modifying therapies; MS, multiple sclerosis; mQoL, mental quality of life; na, not available at baseline; pQoL, physical quality of life; PRSES, perceived relative socioeconomic status; SD, standard deviation; SLEs, stressful life events. χ^2^ and *t*-test. ^*^p < 0.05; ^**^p < 0.01; ^***^p < 0.001

### Associations between SLEs and QoL, moderated by mastery

SLEs were significantly associated with lower mQoL in pwMS with low and moderate mastery, but not with high mastery (Table 2). Group differences were observed beween low and high, and between moderate and high mastery.

**Table 2:**
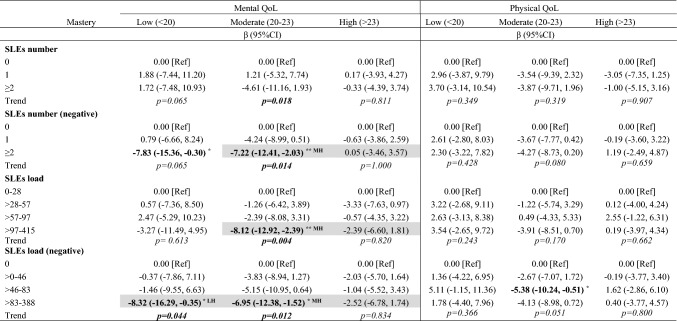
Associations between SLEs and QoL in low, moderate and high mastery

Analysis by multivariable linear regression with interaction terms. Adjusted regression coefficients presented, models adjusted for ongoing symptoms due to recent relapse, sex, age, MS phenotype, disability, relative perceived socioeconomic status, number of comorbidities, fatigue, use of antidepressant, and social support persons. Highlighted cells indicate significant group differences by mastery level (MH = moderate vs. high; LH = low vs. high). Boldface values denote significant association between SLEs and QoL (^*^p < 0.05, ^**^p < 0.01)

CI, confidence interval; QoL, quality of life; SLEs, stressful life events

Experiencing ≥ 2 negatively impacting SLEs was significantly associated with lower mQoL in pwMS with low mastery (β = − 7.83, 95% CI = − 15.36, − 0.30) and moderate mastery (β = − 7.22, 95% CI = − 12.41, − 2.03), corresponding to approximately 8-point and 7-point differences on the MSQoL-54 scale, respectively; no association was observed among pwMS with high mastery. Significant group difference was observed between moderate and high mastery (β_difference_ = 7.66, *p* = 0.04). The top quartile of negative SLEs load was signicantly associated with lower mQoL (β =− 8.12; 95%CI = − 12.92, -2.39) in moderate, but not high mastery, with significant group difference observed (β_difference_ = 7.86; *p* = 0.04).

The top quartile of negative SLEs load was associated with lower mQoL in pwMS with low mastery (β = − 8.32, 95% CI = − 16.29, − 0.35) and moderate mastery (β = − 6.95, 95% CI = − 12.38, − 1.52), respectively. This association was not significant in high mastery; group differences were observed between low and high (β_difference_ = 6.04; *p* = 0.04), and moderate and high (β_difference_ = 7.66, *p* = 0.04) mastery.

No difference in SLEs-mQoL associations were observed between low and moderate mastery. No consistent associations were seen between SLEs number or load and pQoL.

### Associations between SLEs and clinically significant fatigue, moderated by mastery

SLEs were associated with higher odds of fatigue in pwMS with low mastery (Table 3). No associations were observed with moderate or high mastery.

**Table 3:**
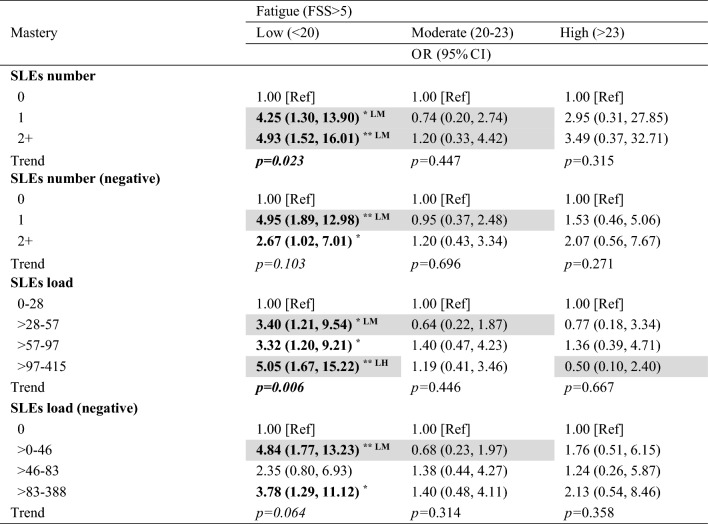
Associations between SLEs and fatigue in low, moderate and high mastery

Analyses by multivariate logistic regression with interaction term. Fatigue model adjusted for ongoing symptoms due to recent relapse, sex, age, MS phenotype, disability, perceived relative socioeconomic status, social support persons, number of comorbidities, depression, and use of anti-fatigue medication. Highlighted cells indicate significant group differences by mastery level (LM = low vs. moderate; LH = low vs. high). Boldface values denote statistical significance (^*^p < 0.05; ^**^p < 0.01)

CI, confidence interval. FSS, fatigue severity scale; QoL, quality of life; SLEs, stressful life event

Experiencing 1 or ≥ 2 SLEs was associated with 4 (OR = 4.25, 95%CI = 1.30, 13.90) and 5 (OR = 4.93, 95%CI = 1.52, 16.01) times higher odds of fatigue, respectively, in pwMS with low mastery. Group difference was observed between low and moderate mastery (OR_difference_ = 5.77–9.85; *p* = 0.04). Experiencing 1 or ≥ 2 negative SLEs was associated with 5 (OR = 4.95, 95%CI = 1.89, 12.98) and 3 (OR = 2.67, 95%CI = 1.02, 7.01) times higher odds of fatigue, respectively, in low mastery. Group difference was observed between low and moderate mastery (OR_difference_ = 7.12; *p* = 0.003) on 1 negative SLEs.

All three levels of total SLEs load were associated with higher odds of fatigue in low mastery. Group differences were observed between low and moderate mastery on > 28–57 total SLEs load (OR_difference_ = 5.50–9.63; *p* = 0.04), and between low and high mastery on > 97–415 total SLEs load (OR_difference_ = 6.34, *p* = 0.03).

Negative SLEs load of > 0–46 and > 83–388 were associated with 5 (OR = 4.84, 95%CI = 1.77, 13.23) and 4 (OR = 3.78, 95%CI = 1.29, 11.12) times higher odds of fatigue in low mastery, respectively. Group difference was observed between low and moderate mastery (OR_difference_ = 6.54, *p* = 0.03) on > 0–46 negative SLEs load.

## Discussion

Living with MS is inherently stressful due to unpredictable and debilitating symptoms, which may increase vulnerability to the impact of life stressors [8]. To our knowledge, this is the first study in pwMS to test mastery as an effect modifier of associations between SLEs exposure and mQoL and clinically significant fatigue. SLE exposure was associated with lower mQoL among pwMS with low or moderate mastery, whereas these associations were attenuated among those with high mastery. High mastery modified associations of SLEs number and load with mQoL, with approximately a 7-point difference in mQoL between pwMS with high versus moderate/low mastery. Mastery also modified associations between SLEs exposure and fatigue, with higher SLEs number and load associated with 3–5 fold higher odds of fatigue among pwMS with low mastery.

Total number of negatively impacting SLEs and higher SLEs load were associated with 7–8 points lower mQoL in pwMS with low and moderate mastery, respectively. This aligns with prior work showing that higher perceived stress is associated with lower mQoL in pwMS and in cancer populations [9, 14]. Associations with mQoL were stronger for negatively impacting SLEs (number and load) than for the overall number of SLEs, likely because negatively appraised events (e.g., death or illness of a family member) exert a greater impact on mental wellbeing than events commonly perceived as positive (e.g., gaining a new family member). Additionally, a difference between low and high mastery was observed for SLEs load, but not SLEs number, suggesting that load may better capture the stress burden of major events. Differences in how studies classify SLEs (negative vs combined positive/negative) and quantify exposure (number vs severity/load) may therefore contribute to heterogeneity in reported associations with health outcomes [9, 13, 14]. Future studies that distinguish negatively impacting SLEs from overall SLEs, as well as number and load of these events, may offer more comprehensive insights.

Associations between the number and load of SLEs, either total or negative, with mQoL were non-significant in pwMS with high mastery. Moderation analyses revealed approximately 7-point group differences in mQoL between pwMS with high mastery compared to pwMS with moderate and low mastery, exceeding the clinically meaningful threshold of points on the MSQoL-54 composite scale [37, 38], indicating that these differences are not only statistically significant but also clinically meaningful for pwMS. This suggests that pwMS who have high mastery are less susceptible to the adverse effects of SLEs. While, to the authors’ knowledge, no prior study has investigated the protective role of mastery in pwMS, the findings are consistent with a study showing high mastery in people with HIV mitigated the negative effects of SLEs number on mQoL by 11% [25]. Individuals with high mastery may accept life stressors and may engage in more adaptive coping strategies, such as ‘planning’ and ‘positive reframing’, and less likely to use ‘denial’, ‘self-blame’, and ‘substance use’, when facing challenging situations compared to those with lower mastery [24, 39–41]. This may explain how mastery buffers the adverse impact of SLEs on mQoL.

We found no consistent association between SLEs number or load and pQoL across mastery levels, aligning with previous studies in chronic lymphocytic leukemia and breast cancer patients [14, 42]. However, our results contradict more recent studies in pwMS reporting that perceived stress is associated with lower pQoL domains [9]. Other studies have also reported negative associations between SLEs and pQoL in cancer patients [13, 25]. These inconsistent findings may be due to variations in the analytical approaches employed, tools used to measure QoL and mastery, or the possibility that specific SLEs are associated with domains of pQoL. For example, while Kołtuniuk and colleagues (2021) assessed the univariate relationships between perceived life stress and pQoL, we adjusted for a range of relevant clinical and demographics factors for a robust assessment of SLEs-pQoL associations [9]. Additionally, our study focused on SLEs experienced in the past 12 months, whereas previous studies included illness-specific (e.g., reaction to treatment) or long-acting events (e.g., childhood adversity) [13, 14] that may have stronger impact on physical functioning [43].

While studies in pwMS have reported 7–15% higher fatigue when exposed to SLEs [17, 18], previously, we found non-significant associations between SLEs and fatigue in this population of pwMS [19]. In the curent study, we showed a negative association between SLEs and fatigue in pwMS, when level of mastery is considered. SLEs number and load were associated with higher odds of clinically significant fatigue in pwMS with low mastery (ORs approximately 3–5), but not in those with moderate or high mastery. Consistent with our findings, Swanepoel and colleagues (2020) also revealed higher fatigue among pwMS with lower mastery when exposed to SLEs [18]. Mastery may influence the association between SLEs on fatigue through various pathways. Firstly, pwMS with low mastery may experience a heightened stress response when experiencing SLEs, including elevated stress hormones, poor quality sleep, and alterations in energy metabolism, all contributing to increased fatigue [23, 44, 45]. PwMS with low mastery tend to employ maladaptive coping strategies, such as detached/avoidant coping which are associated with increased fatigue [40, 41, 46], while pwMS with high mastery are more likely to engage in healthy lifestyle behaviours, such as physical activity [41, 47], which may mitigate fatigue in stressful situations [48]. While associations between SLEs and fatigue are complex; mastery may play a protective role for stress management.

To our knowledge, this study provides novel evidence that mastery may buffer adverse associations between SLEs exposure and mQoL and fatigue in pwMS. We previously reported in this populaiton, meditation practice is associated with higher self-mastery [49], and that predictors of 5-year high self-mastery trajectories include socieconomic status, depression and fatigue, as well as diet quality and regular physical activity (Nag et al., under review); these all being potential intervention targets to improve self-mastery. Strengths of our study include the large international sample, multivariable models with adjustment for a broad set of relevant demographic and clinical confounders, and detailed assessment of SLEs exposure using both event number and load, enabling a more nuanced evaluation of stress burden. The primary limitation is healthy participant bias, with higher proportion of study participants having a university education, more support, better clinical characteristics, and a higher mastery score; therefore, the generalisability of our findings may be limited. Although confounders were adjusted for, future studies in more diverse pwMS samples are needed to substantiate these findings. In addition, most pwMS reported experiencing 1–2 SLEs in the past 12 months and illness-specific stressors were not captured, which may have underestimated overall stress exposure and contributed to the non-significant associations observed for pQoL. Future studies using tools that include disease-specific stressors and biomarkers of stress may provide additional insight.

## Conclusions

These findings highlight mastery as a potentially modifiable psychological resource that may enhance resilience to stress in pwMS. Interventions targeting mastery—such as those incorporating behavioural, cognitive, and lifestyle approaches including physical activity and meditation—may represent promising strategies to mitigate stress-related decrements in wellbeing. Given the cross-sectional design, longitudinal and interventional studies are needed to establish causality and determine whether enhancing mastery leads to sustained improvements in QoL and fatigue. Future research should also explore mechanisms linking mastery, stress appraisal, and health behaviours to inform targeted interventions.

## Data Availability

De-identified aggregate data that support the findings of this study are available from NN or JR upon reasonable request.
